# Chronic disease and multimorbidity in the Chinese older adults’ population and their impact on daily living ability: a cross-sectional study of the Chinese Longitudinal Healthy Longevity Survey (CLHLS)

**DOI:** 10.1186/s13690-024-01243-2

**Published:** 2024-02-01

**Authors:** Ye Chen, Huixia Ji, Yang Shen, Dandan Liu

**Affiliations:** Department of Occupational Disease, Nanjing Prevention and Treatment Center for Occupational Diseases, Nanjing, Jiangsu China

**Keywords:** Older adults, Chronic disease, Multimorbidity, Activities of daily life, Instrumental activities of daily life

## Abstract

**Background:**

Owing to an increase in life expectancy, it is common for the older adults to suffer from chronic diseases that can result in disability and a low quality of life. This study aimed to explore the influence of chronic diseases and multimorbidities on activities of daily living (ADLs) and instrumental ADLs (IADLs) in an older Chinese population.

**Methods:**

Based on the Chinese Longitudinal Healthy Longevity Survey (2018), 9,155 older adults aged 65 years and above were included in the study. A self-administered questionnaire was used to collect information on demographic characteristics, chronic diseases, ADLs, and IADLs. The impact of factors affecting ADL and IADL impairment in older adults was analysed using binary logistic regression.

**Results:**

In total, 66.3% participants had chronic diseases. Hypertension, heart disease, arthritis, diabetes and cerebrovascular disease were among the top chronic diseases. Of these, 33.7% participants had multimorbidities. The most common combination of the two chronic diseases was hypertension and heart disease (11.2%), whereas the most common combination of the three chronic diseases was hypertension, heart disease, and diabetes (3.18%). After categorising the older adults into four age groups, dementia, visual impairment, and hearing impairment were found to be more prevalent with increasing age. The prevalence of hypertension, heart disease, cerebrovascular disease, gastrointestinal ulcers, arthritis and chronic nephritis gradually increased with age until the age of 75 years, peaked in the 75–84 years age group, and then showed a decreasing trend with age. Multimorbidity prevalence followed a similar pattern. Regression analysis indicated that the increase in age group and the number of chronic diseases independently correlated with impairments in ADL as well as IADL. Additionally, gender, physical activity, educational background, obesity, depressive symptoms, and falls also had an impact on ADLs or IADLs.

**Conclusion:**

Chronic diseases and multimorbidities are common in older adults, and it is important to note that aging, multimorbidity, obesity, and unhealthy lifestyle choices may interfere with ADLs or IADLs in older adults. Therefore, it is imperative that primary healthcare providers pay special attention to older adults and improve screening for multimorbidity and follow-up needs.

**Table:** 

Text Box 1. Contributions to the literature
The CLHLS study indicated that chronic diseases and multimorbidities increase with age, but they gradually decline after they peak between 75 and 84 years of age. It is believed that the age group of 75–84 years is critical for the longevity of the older adults.
Gender, physical activity, educational background, obesity, depression, and falls were also associated with ADLs or IADLs.
Having regular medical checkups by the government will assist in detecting potential risks of incapacity early on and preventing them from occurring, making it easier for the elderly to take care of themselves on a daily basis.

## Background

Population aging is a major challenge for most countries. According to the National Aging Development Bulletin (2021), the older adults’ population of China was 200 million in 2021, accounting for 14.2% of the total population. With an increase in life expectancy, healthy aging has become a goal for modern older adults in China. Furthermore, among the older adults, activities of daily living (ADL) and instrumental ADL (IADL) are important indicators of the basic ability to live independently and socially and are considered potential indicators of the quality of life of the individual.

As a result of social development and an increase in life expectancy, chronic diseases, such as cardiovascular diseases and malignant tumours, have become more prevalent. Chronic patients often have  more than one chronic disease. In 2008, the World Health Organisation defined multimorbidity as the presence of two or more coexisting chronic diseases. Considering the inconsistency in chronic diseases, there is no standard definition for multimorbidity. The effects of chronic diseases and multimorbidities include disability, reduced quality of life, shorter life expectancy, and greater need for healthcare resources [1]. Additionally, medical workers have gradually realised that single-disease guidelines cannot be applied to older adults, and diagnosis and treatment have gradually evolved to become patient-centred rather than disease-centred. Over the past few years, research on chronic disease and multimorbidity has gradually gained momentum in China [2]. Therefore, identifying multimorbidity and high-risk factors that adversely affect the quality of life of older individuals is a critical public health concern.

Consequently, based on the Chinese Longitudinal Healthy Longevity Survey (CLHLS)-(2018), we analysed the demographic variables, chronic diseases, depressive symptoms, falls and ADL and IADL status of older adults aged over 65 years, as well as factors influencing ADL and IADL. This study aimed to improve quality of life and promote healthy aging in the older population.

## Methods

### Data sources

The study population was derived from the 2017–2018 survey data in the CLHLS program, which used multi-stage whole cluster sampling in 23 Chinese provinces (Beijing, Tianjin, Chongqing, Shanghai, Anhui, Fujian, Guangdong, Guangxi, Hainan, Hubei, Hunan, Henan, Hebei, Heilongjiang, Liaoning, Jiangxi, Jiangsu, Jilin, Shandong, Shanxi, Shanxi, Sichuan and Zhejiang), 501 counties (districts) were selected as the study site using simple random sampling method, and all centenarians in the selected counties (districts) who voluntarily participated in the survey were surveyed, and 1 each of 70–79, 80–89 and 90–99 year olds living near the elderly were matched according to the gender of the elderly, and 0.5 of 65–69 year olds were surveyed. A total of 15,874 elderly were surveyed in 2017–2018, and the specific study design is detailed in references [3, 4]. A uniformly trained investigator, nurse, or medical senior conducted face-to-face interviews and physical examinations for each participant. The CLHLS was approved by the Ethics Committee of the Peking University (No: IRB0000105213074), and written informed consent was obtained from all participants (or their representatives). The datasets supporting the conclusions of this study are available in the Peking University Open Research Data repository, unique persistent identifier, and hyperlink to the datasets at [https://opendata.pku.edu.cn/dataset.xhtml?persistentId=doi:10.18170/DVN/WBO7LK].

### Inclusion criteria

**Fig. 1:**
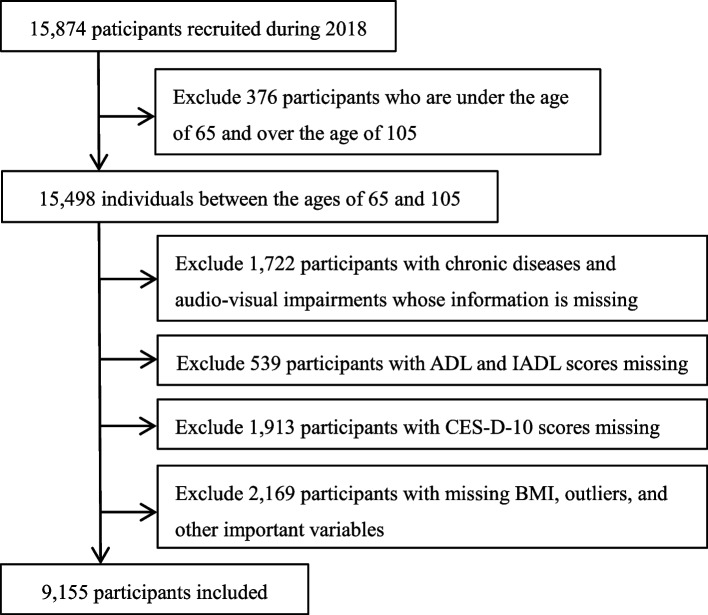
Flow chart of participants included in analysis

According to a quality assessment of older adults’ information declarations, the information provided by older adults aged over 105 years is not of high quality [5]. Older participants aged 65–105 years were included in this study. The exclusion criteria were missing data on ADL, IADL, residence, education, smoking, physical activity, chronic disease, depression, falls, body mass index (BMI), and outliers. A total of 9,155 participants were enrolled in this study. A flowchart of the screening process is shown in Fig. 1.

### Questionnaire

A physical self-care scale was administered as part of the questionnaire. The scale contained six items, including bathing, dressing, toileting, indoor activities, bowel movement control, and eating. If the individual was able to complete the scale independently, then ADL was normal; if there were difficulties in any activity, then ADL was impaired. An individual’s ability to perform an item on the IADL scale was impaired if any of these tasks was difficult to perform, including going out, shopping, cooking, washing, walking 2 km, carrying 5 kg of goods, squatting, or using public transportation.

A brief version of the Center for Epidemiologic Studies Depression Scale (CESD-10) measures depressive symptoms in older individuals [6]. A depression score was calculated by summing the scores for the 10 items, which ranged from 1 to 5. Generally, the higher the score, the more severe the depression. Depressive symptoms were defined as a score greater than 20.

### Evaluation indicators

①Fifteen types of chronic diseases were included in this study, including hypertension, diabetes, heart disease, cerebrovascular disease (CVD), bronchitis, emphysema/pneumonia/asthma (respiratory disease), malignant tumours, gastrointestinal ulcers, Parkinson's disease, pressure sores, arthritis, dementia, cholecystitis/cholelithiasis (biliary tract disease), dyslipidaemia, rheumatoid disease, and chronic nephritis, and two types of poor health status, visual impairment and hearing impairment, were included in the study. Chronic disease was based on self-reported ‘whether you have this disease or bad condition’. Multimorbidity was defined as the simultaneous presence of more than one chronic illness at the same time. The term ‘visual or hearing impairment’ refers to a self-reported inability to see test objects or hear interrogator questions during interview [7]. ②A BMI less than 18.5 kg/m^2^ indicated underweight status, 18.5–24.0 kg/m^2^ indicated normal weight, 24–28.0 kg/m^2^ indicated overweight, and more than 28.0 kg/m^2^ indicated obese. ③Demographic characteristics, including age, sex, place of residence, and attendance at school, were considered. ④ Living habits: Smoking or not; Is any physical activity involved?; ⑤ ADL and IADL: Are they impaired?; ⑥Depression: The score of brief version of CESD-10 more than 20; ⑦Falls: Have you fallen in the past year?

### Statistical analysis

Older individuals were divided into four age groups: 65–74 years, 75–84 years, 85–94 years, and 95–105 years. Categorical data were analysed using chi-square test. The Cochran–Armitage trend test was used to determine upward or downward trends in demographic information, depressive symptoms, falls, BMI, chronic diseases, and the prevalence of multimorbidity. The Apriori algorithm was used to analyse associations between chronic diseases. Regression analysis was performed on the dependent variable, ADL or IADL impairment, to determine whether it was influenced by demographic information, chronic diseases, BMI, etc. The R package (v4.3.1) was used for statistical analysis. α = 0.05.

## Results

### Basic characteristics

The median age of the study population was 82 (74, 91) years. There were 4,289 men (46.8%) and 4,866 women (53.2%) in this study; of these, 66.3% (6,071/9,115) had chronic diseases, and 33.8% (3,086/9,115) had multimorbidities. Individuals with impaired ADL and IADL accounted for 17.1% (1,566/9,115) and 60.1% (5,501/9,115), respectively. About 52.9% (4,840/9,115) of individuals were diagnosed with depressive symptoms according to the CESD-10, and 21.5% (1,944/9,115) of individuals had fallen in the past year. The demographic data, chronic diseases, depressive symptoms, falls, and ADL and IADL impairments are shown in Table 1. The proportion of men, education, physical activity, smoking, no depression, no falls and normal proportion of ADL and IADL gradually decreased as the age group increased, with a chi-square trend test *p* < 0.05.

**Table 1:** Comparison of basic characteristics of the elderly in different age groups

Variants	All	65–74 years	75–84 years	85–94 years	95–105 years	*p*	*p* for trend
	*N* = 9155	*N* = 2519	*N* = 2735	*N* = 2452	*N* = 1449		
Sex						< 0.001	< 0.001
Male	4289 (46.8%)	1268 (50.3%)	1332 (48.7%)	1195 (48.7%)	494 (34.1%)		
Female	4866 (53.2%)	1251 (49.7%)	1403 (51.3%)	1257 (51.3%)	955 (65.9%)		
Residence						0.052	0.007
Rural	3814 (41.7%)	1100 (43.7%)	1146 (41.9%)	989 (40.3%)	579 (40.0%)		
City & Town	5341 (58.3%)	1419 (56.3%)	1589 (58.1%)	1463 (59.7%)	870 (60.0%)		
Education						< 0.001	< 0.001
No	3915 (42.8%)	535 (21.2%)	1055 (38.6%)	1339 (54.6%)	986 (68.0%)		
Yes	5240 (57.2%)	1984 (78.8%)	1680 (61.4%)	1113 (45.4%)	463 (32.0%)		
Physical activity						< 0.001	< 0.001
No	5839 (63.8%)	1396 (55.4%)	1567 (57.3%)	1674 (68.3%)	1202 (83.0%)		
Yes	3316 (36.2%)	1123 (44.6%)	1168 (42.7%)	778 (31.7%)	247 (17.0%)		
Smoking						< 0.001	< 0.001
No	7670 (83.8%)	1958 (77.7%)	2280 (83.4%)	2114 (86.2%)	1318 (91.0%)		
Yes	1485 (16.2%)	561 (22.3%)	455 (16.6%)	338 (13.8%)	131 (9.04%)		
BMI						< 0.001	< 0.001
Normal	4751 (52.1%)	1172 (46.7%)	1414 (51.9%)	1372 (56.1%)	793 (54.8%)		
Underweight	1315 (14.4%)	134 (5.34%)	313 (11.5%)	436 (17.8%)	432 (29.8%)		
Overweight	2291 (25.1%)	908 (36.2%)	732 (26.9%)	476 (19.5%)	175 (12.1%)		
Obese	770 (8.44%)	295 (11.8%)	264 (9.70%)	163 (6.66%)	48 (3.31%)		
Number of Chronic disease						< 0.001	< 0.001
0	3084 (33.7%)	793 (31.5%)	743 (27.2%)	822 (33.5%)	726 (50.1%)		
1	2985 (32.6%)	830 (32.9%)	921 (33.7%)	800 (32.6%)	434 (30.0%)		
2	1632 (17.8%)	468 (18.6%)	537 (19.6%)	452 (18.4%)	175 (12.1%)		
3	769 (8.40%)	237 (9.41%)	256 (9.36%)	209 (8.52%)	67 (4.62%)		
≥ 4	685 (7.48%)	191 (7.58%)	278 (10.2%)	169 (6.89%)	47 (3.24%)		
ADL impairment						< 0.001	< 0.001
No	7589 (82.9%)	2437 (96.7%)	2539 (92.8%)	1876 (76.5%)	737 (50.9%)		
Yes	1566 (17.1%)	82 (3.26%)	196 (7.17%)	576 (23.5%)	712 (49.1%)		
IADL impairment						< 0.001	< 0.001
No	3654 (39.9%)	1915 (76.0%)	1254 (45.9%)	416 (17.05%)	69 (4.76%)		
Yes	5501 (60.1%)	604 (24.0%)	1481 (54.1%)	2036 (83.0%)	1380 (95.2%)		
Depression						< 0.001	< 0.001
No	4315 (47.1%)	1351 (53.6%)	1279 (46.8%)	1073 (43.8%)	612 (42.2%)		
Yes	4840 (52.9%)	1168 (46.4%)	1456 (53.2%)	1379 (56.2%)	837 (57.8%)		
Falls						< 0.001	< 0.001
No	7110 (78.5%)	2096 (84.0%)	2120 (78.4%)	1872 (77.1%)	1022 (71.6%)		
Yes	1944 (21.5%)	400 (16.0%)	583 (21.6%)	556 (22.9%)	405 (28.4%)		

*BMI* Body mass index, *ADL* Activities of daily living, *IADL* Instrumental activities of daily living

### Prevalence of chronic diseases

**Fig. 2:**
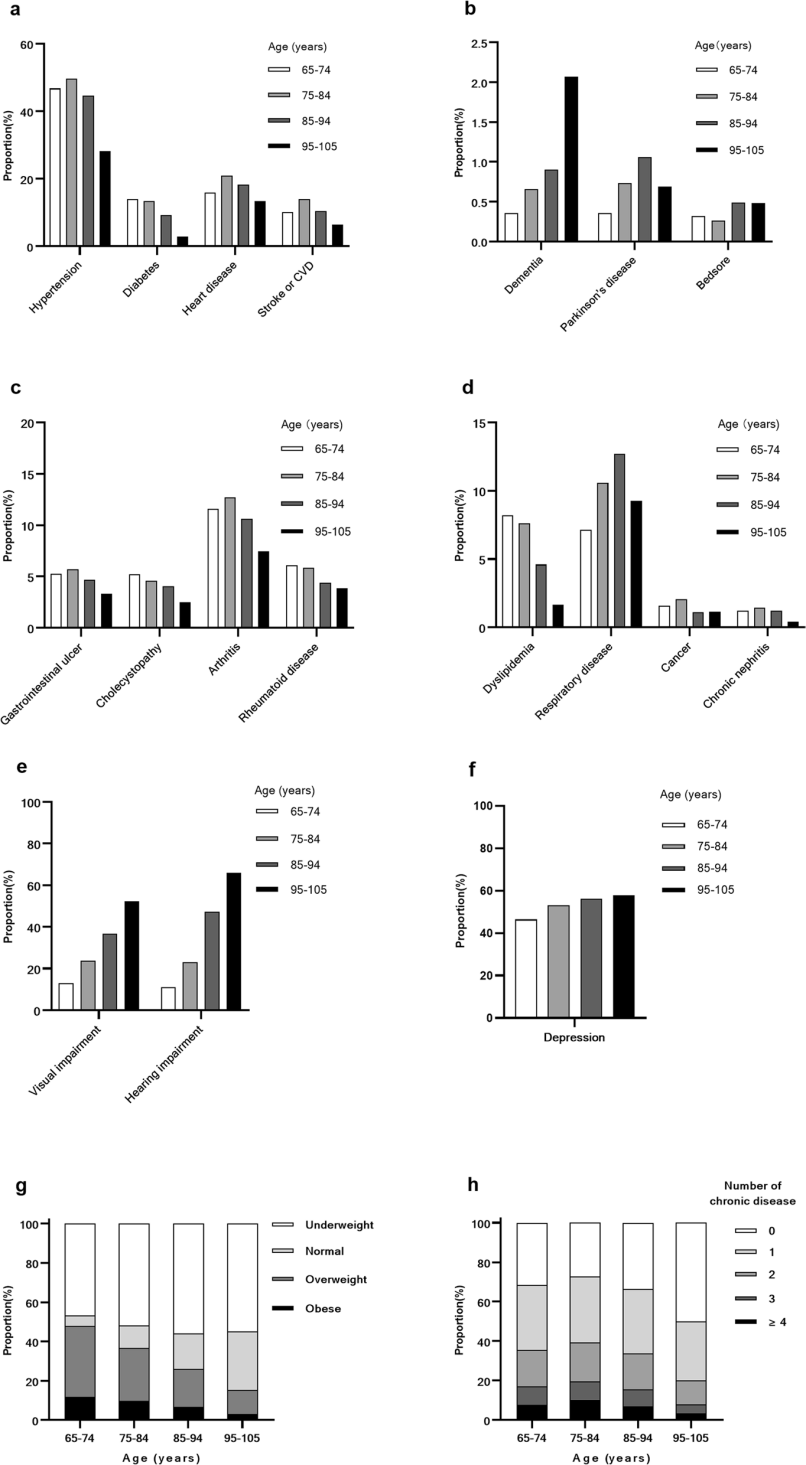
Proportion of chronic diseases, visual impairments, hearing impairments, depression, BMI status and number of chronic diseases according to their age group

Hypertension (44.1%), heart disease (17.6%), arthritis (11.0%), diabetes (10.8%), CVD (10.7%), and respiratory disease (10.0%) had the highest prevalence rates. The prevalence in each age group is shown in Table 2 and Fig. 2. With advancing age, the overall prevalence rates of hypertension, diabetes, CVD, respiratory disease, gastrointestinal ulcers, biliary tract diseases, arthritis, rheumatoid disease, chronic nephritis and hyperlipidaemia showed a downward trend (*p* < 0.05). However, the prevalence of hypertension, heart disease, CVD, gastrointestinal ulcers, chronic nephritis, and arthritis increased gradually, peaked at the age of 75–84 years, and then decreased thereafter. Dementia, depression, visual impairment, and hearing impairment generally increased with age (*p* < 0.05). As the age group increased, the proportion of underweight and normal weight individuals gradually increased, whereas the proportion of overweight and obese individuals gradually decreased, as shown in Table 2 and Fig. 2. (*p* for trend < 0.05), respectively.

**Table 2:** Chronic diseases among the elderly by age group

	All	65–74 years	75–84 years	85–94 years	95–105 years	*p*	*p* for trend
	*N* = 9155	*N* = 2519	*N* = 2735	*N* = 2452	*N* = 1449		
Hypertention	4036 (44.1%)	1177 (46.7%)	1357 (49.6%)	1094 (44.6%)	408 (28.2%)	< 0.001	< 0.001
Diabetes	985 (10.8%)	353 (14.0%)	365 (13.3%)	226 (9.22%)	41 (2.83%)	< 0.001	< 0.001
Heart disease	1615 (17.6%)	401 (15.9%)	571 (20.9%)	449 (18.3%)	194 (13.4%)	< 0.001	0.094
CVD	984 (10.7%)	254 (10.1%)	381 (13.9%)	256 (10.4%)	93 (6.42%)	< 0.001	< 0.001
Respiratory disease	917 (10.0%)	180 (7.15%)	291 (10.6%)	312 (12.7%)	134 (9.25%)	< 0.001	< 0.001
Cancer	141 (1.54%)	40 (1.59%)	57 (2.08%)	27 (1.10%)	17 (1.17%)	0.021	0.065
Gastrointestinal ulcer	452 (4.94%)	133 (5.28%)	156 (5.70%)	115 (4.69%)	48 (3.31%)	0.006	0.004
Parkinson’s disease	65 (0.71%)	9 (0.36%)	20 (0.73%)	26 (1.06%)	10 (0.69%)	0.033	0.043
Bedsore	34 (0.37%)	8 (0.32%)	7 (0.26%)	12 (0.49%)	7 (0.48%)	0.459	0.216
Arthritis	1009 (11.0%)	293 (11.6%)	348 (12.7%)	260 (10.6%)	108 (7.45%)	< 0.001	< 0.001
Dementia	79 (0.86%)	9 (0.36%)	18 (0.66%)	22 (0.90%)	30 (2.07%)	< 0.001	< 0.001
Cholecystopathy	393 (4.29%)	132 (5.24%)	126 (4.61%)	99 (4.04%)	36 (2.48%)	< 0.001	< 0.001
Dyslipidemia	552 (6.03%)	207 (8.22%)	208 (7.61%)	113 (4.61%)	24 (1.66%)	< 0.001	< 0.001
Rheumatoid disease	478 (5.22%)	154 (6.11%)	160 (5.85%)	108 (4.40%)	56 (3.86%)	0.002	< 0.001
Chronic nephritis	106 (1.16%)	31 (1.23%)	39 (1.43%)	30 (1.22%)	6 (0.41%)	0.03	0.041
Hearing impairment	3022 (33.0%)	279 (11.1%)	630 (23.0%)	1157 (47.2%)	956 (66.0%)	< 0.001	< 0.001
Visual impairment	2634 (28.8%)	325 (12.9%)	649 (23.7%)	903 (36.8%)	757 (52.2%)	< 0.001	< 0.001
Depression	4840 (52.9%)	1168 (46.4%)	1456 (53.2%)	1379 (56.2%)	837 (57.8%)	< 0.001	< 0.001

*CVD* Cardiovascular disease

### Multimorbidity

Aside from visual and hearing impairments, the proportion of people with one, two, three, four, or more chronic diseases first increased with age, reached a peak in the 75–84 years age group, and then gradually decreased, as shown in Table 1 and Fig. 2. According to association analysis (Table 3), the most common combination of two chronic diseases in the multimorbidity population was hypertension + heart disease in 1,026 cases (11.2%), followed by hypertension + diabetes in 730 cases (7.97%) and hypertension + CVD in 637 cases (6.96%); the most common combination of three chronic diseases was hypertension + heart disease + diabetes in 291 cases (3.18%), followed by hypertension + heart disease + diabetes in 266 cases (2.91%) and hypertension + diabetes + CVD in 162 cases (1.77%).

**Table 3:** Patients with the most common combination of two or three chronic diseases

Dyads of morbidity			Triads of morbidity		
n(%)	LHS	RHS	n(%)	LHS	LHS
1026(11.21)	Hypertention = "T"	Heart disease = "T"	291(3.18)	Hypertention = "T", Heart disease = "T"	Diabetes = "T"
730(7.97)	Hypertention = "T"	Diabetes = "T"	266(2.91)	Hypertention = "T", Heart disease = "T"	CVD = "T"
637(6.96)	Hypertension = "T"	CVD = "T"	162(1.77)	Hypertention = "T", Diabetes = "T"	CVD = "T"

### Factors influencing ADL and IADL impairment

**Fig. 3:**
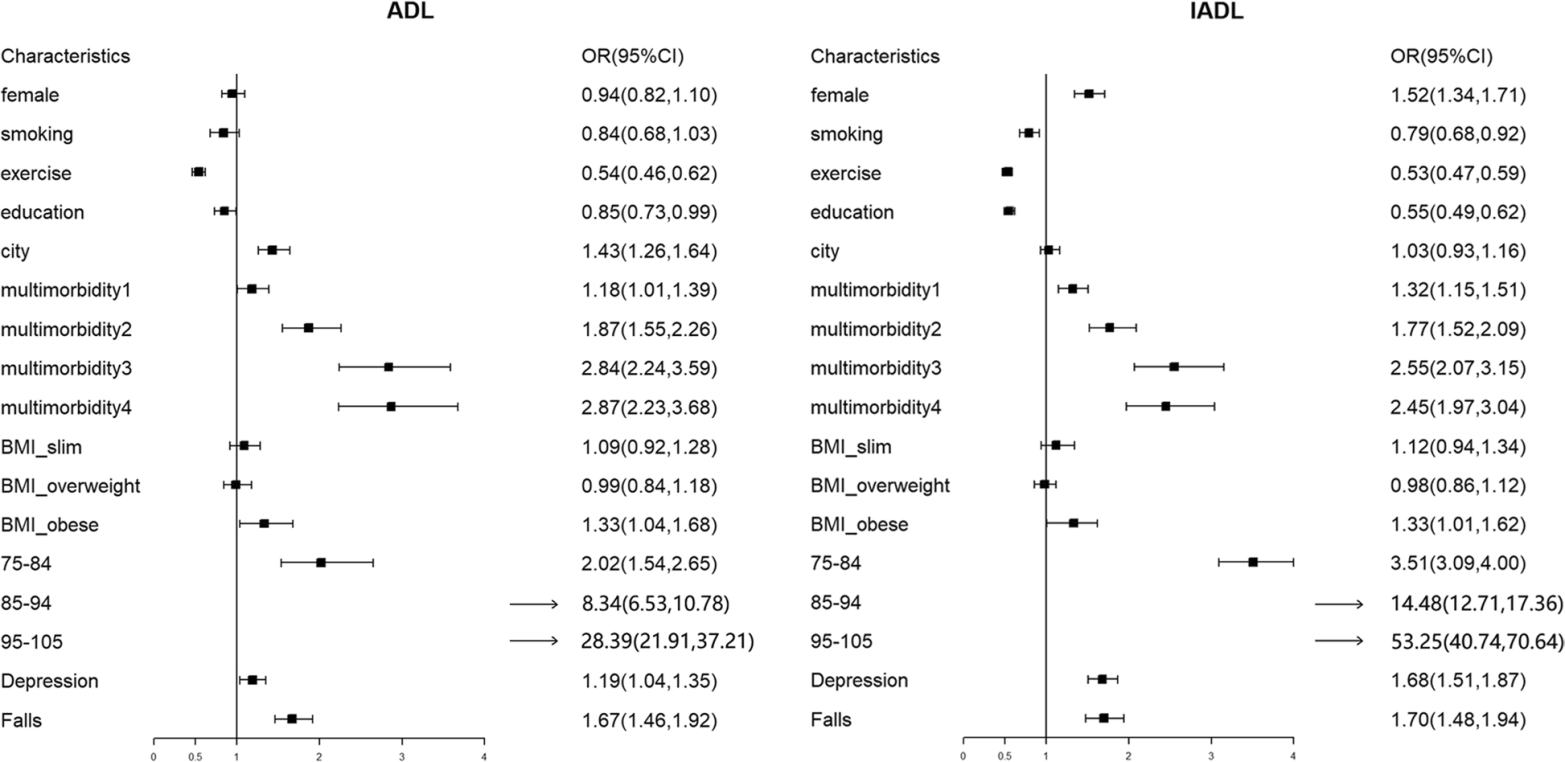
Influencing factors related to ADL and IADL impairment in the elderly

Considering whether ADL or IADL was impaired as the dependent variable, model 1 included the number of chronic diseases and age group as the independent variables. Based on model 1, model 2 included sex, smoking status, physical activity, education, residence, depression, and falls. Based on model 2, model 3 incorporated BMI and performed a binary logistic regression analysis. As shown in Table 4 and Fig. 3, with the increase in the number of chronic diseases and increase in the age group, there was a gradual increase in the odds ratio value, with *p* for trend < 0.05. After considering all variables, physical activity, education, no depression and no falls were found to be protective factors for ADL and IADL. Taking the normal BMI (18.5–23.9 kg/m^2^) as a reference, obesity were risk factors for ADL and IADL impairments. City of residence was a risk factor for impaired ADL, whereas female sex was a risk factor for impaired IADL.

**Table 4:** Age, chronic disease and the odds ratio of ADL and IADL

ADL	Model 1		Model 2		Model 3	
	OR(95%*CI*)	*p for trend*	OR(95%*CI*)	*p for trend*	OR(95%*CI*)	*p for trend*
Age		< 0.001		< 0.001		< 0.001
65–74 years (Ref)						
75–84 years	2.22(1.71–3.212.91)		2.03(1.55–2.66)		2.02(1.54,2.65)	
85–94 years	9.70(7.67–12.43)		8.29(6.51–10.69)		8.34(6.53,10.78)	
95–105 years	36.23(28.39–46.80)		28.19(21.86–36.77)		28.39(21.91,37.21)	
Chronic disease		< 0.001		< 0.001		< 0.001
0 (Ref)						
1	1.16(0.99–1.35)		1.18(1.01–1.38)		1.18(1.01,1.39)	
2	1.90(1.59–2.27)		1.88(1.56–2.26)		1.87(1.55,2.26)	
3	2.94(2.35–3.68)		2.84(2.26–3.59)		2.84(2.24,3.59)	
≥ 4	3.05(2.40–3.87)		2.91(2.27–3.71)		2.87(2.23,3.68)	
IADL	Model 1		Model 2		Model 3	
	OR(95%*CI*)	*p for trend*	OR(95%*CI*)	*p for trend*	OR(95%*CI*)	*p for trend*
Age		< 0.001		< 0.001		< 0.001
65–74 years (Ref)						
75–84 years	3.76(3.33–4.23)		3.51(3.09–3.99)		3.51(3.08–4.00)	
85–94 years	16.72(14.51–19.29		14.89(12.79–17.38)		14.84(12.71–17.36)	
95–105 years	76.12(59.00–99.75)		53.44(41.02–70.65)		53.25(40.73–70.64)	
Chronic disease		< 0.001		< 0.001		< 0.001
0 (Ref)						
1	1.25(1.11–1.42)		1.31(1.15–1.50)		1.32(1.15–1.51)	
2	1.76(1.52–2.05)		1.78(1.52–2.09)		1.77(1.51–2.09)	
3	2.54(2.09–3.10)		2.59(2.11–3.19)		2.55(2.07–3.14)	
≥ 4	2.34(1.92–2.87)		2.41(1.95–2.99)		2.45(1.97–3.04)	

*OR* Odds ratio, Model 1was adjusted for age and number of chronic disease; Model 2 was adjusted for variables in Model 1 plus sex, smoking, residence, physical activity and education. Model 3 was adjusted for variable in Model 2 plus BMI

## Discussion

Based on data from the 2018 cross-sectional survey of the CLHLS, this study explored the relationship between chronic disease, multimorbidity, and impairment of ADL and IADL among individuals aged 65–105 years. The overall incidences of ADLs and IADLs in the older adults’ population were 17.1% and 60.1%, respectively, with a gradual increase in impairment with age. It should be noted that the ADL scale used in this study represents basic living ability, whereas the IADL scale reflects the independent living ability and social function of the older adults, which refers to a higher quality of life. Thus, IADL damage is more apparent in older individuals.

According to a meta-analysis of 56 studies in China, the prevalence of ADL disability was 20.5% (95%CI: 17.7–23.3%); older individuals (80 + years) had a significantly higher ADL disability rate than younger elders (30.0%, 95% CI:26.2–33.9%) [8]. A study by South Korean researchers found that in 2020, ADL and IADL limitations were 6.14% and 15.49%, respectively. In the 85 + age group, 7.37% of individuals had severe disabilities for ADLs and 12.06% for IADLs. A high rate of mortality was also observed among people who were undereducated, underweight, inactive, depressed, and suffering from three or more chronic diseases [9]. A study of 49,732 older adults in Japan showed that participation in cultural leisure activities may contribute to improved IADL scores. In addition, the benefits that may be gained from social networks may boost social engagement and enhance health [10]. Impairment of ADL and IADL prevalence rates were not compared because they are assessed differently in each country. However, the prevalence of ADL disabilities increased with age, while IADL disabilities tend to occur earlier in life.

Data from 23 Chinese provinces (municipalities) were used in this study. Chronic diseases with a high prevalence in the older population included hypertension (44.1%), heart disease (17.6%), arthritis (11.0%), diabetes (10.8%), CVD (10.7%), and respiratory system disease (10.0%). This finding is consistent with the results of the recent Survey of Health, Aging, and Retirement in Europe, Swedish VAL data, and a meta-analysis of eight Asian studies [11–13]. Recently, chronic disease patterns in China have gradually evolved into those associated with cardiovascular, cerebrovascular, and metabolic diseases.

The findings of the study indicate that chronic diseases are prevalent in 66.3% of the older adults, and multimorbidity is prevalent in 33.7% of the older adults. As shown in Fig. 2, the prevalence of diabetes gradually decreased from the age of 65 years, indicating that the effects of diabetes on life expectancy began at least at that age or earlier. Many older individuals suffer from hypertension, heart disease, CVD, gastrointestinal ulcers, and arthritis, which first gradually increase as they grow older, peak in the 75–84 years age group, and then gradually decline with age. Figure 2 also illustrates the peak prevalence of chronic diseases and multimorbidities in the 75–84 years age group, followed by a gradual decline with age. Several scholars have studied the same database and found that the 75–79 years group is the key age group in which the quality of life of the older adults can change from good to poor, and that 42.58% of the older adults over the age of 100 years are able to take care of themselves [14]. Notably, the 2015 data from the China Health and Retirement Longitudinal Study also presented similar findings. The prevalence of multimorbidities peaked between the ages of 70–80 years and then gradually decreased in the following decades [15]. The results of a cross-sectional study conducted in Taiwan in 2013 revealed a peak prevalence of multimorbidity in the 80–89 years age group, followed by a gradual decline thereafter [16]. This phenomenon appears to be the result of a survivor bias. Theoretically, chronic diseases are more prevalent with aging, but they significantly affect the life expectancy of the older adults, and only healthy older individuals can survive till older age. This corresponded to an average life expectancy of 77 years in China in 2018. With age, the prevalence of dementia, and audiovisual impairment increases; however, they do not have a significant impact on life expectancy, as these disorders may be compensated for by family or institutional support. Other studies have shown that the incidence of chronic diseases gradually increases with age in countries with better medical conditions, such as Germany [17–19]. Some of this may be attributed to the relatively good medical conditions and complete chronic disease registration system in these countries. Differences in the types of multimorbidities, age stratification, awareness rate of chronic diseases, and even some older people who have limited mobility and cannot complete the survey result in different outcomes. Despite the heterogeneous results of various studies, the prevalence of chronic diseases and multimorbidity is expected to increase with economic development and population aging.

Analysis of this association revealed that hypertension, diabetes, CVD, and heart disease were the most common patterns of multimorbidity. In line with other studies on multimorbidity patterns [11, 13], two or three multimorbidities with the strongest associations were metabolic-related disorders, followed by degenerative conditions. The study was moderately comparable, although there were differences in the samples and chronic diseases. Several factors may contribute to the association between these multimorbidities, including the underlying aetiology, exposure to risk factors before onset, and the involvement of common intermediate metabolites in pathogenesis [20].

Despite the conclusion of this study that smoking is a protective factor against IADL, since 1957 when the British Medical Research Council reported an increase in lung cancer related to smoking, an increasing amount of evidence has led to the conclusion that smoking is harmful to health. In addition to causing lung cancer, cardiovascular disease, and CVD, smoking contributes to a serious reduction in life expectancy and death rates [21–24]. The prevalence of dementia and hemiplegia increases gradually with age, making smoking extremely difficult for them. As defined now, smoking may not cover the full range and dose of tobacco exposure, and smoking cessation may underestimate smoking's health risk [21]. In 2022, some researchers published an article in *Nature Genetics* stating that the mutation rates of single nucleotide variations and small fragment insertions, and deletions were significantly higher in smokers than in the non-smoker population; however, these mutations tended to be stable after 23 packs of smoking, which was thought to be related to DNA loss repair enhancement [25]. Based on a comprehensive review of several studies [11, 26–28], we still believe that smoking is injurious to health.

In older patients, being obese is generally considered detrimental to health. According to the Finnish and British Biobank cohort studies, obese and overweight older patients have a much greater risk of developing multimorbidities [29]. A Korean study involving more than 11,000 adults found that those who were overweight or obese were twice as likely to develop hypertension, dyslipidaemia, diabetes, and osteoarthritis compared to those with a normal BMI [30]. Additionally, these diseases are highly correlated, with the presence of one disease increasing the risk of the other, and many of their associations are bidirectional. Whether obese individuals have a single chronic disease or a combination of chronic diseases [11, 28], obesity increases mortality in these populations. Obesity is related to impairments in ADLs and IADLs. As life expectancy increases, the proportion of people with a BMI between 18.5–24.0 kg/m^2^ increases with age, indicating that their quality of life may be maintained, and they can reach an older age group.

Living in rural areas and exercising regularly reduced the risk of ADL impairment. As rural life does not offer the same convenience as urban living and health care conditions are generally poor, rural residents must be able to exercise greater self-care skills to remain healthy. Thus, healthier older adults are more likely to survive because of the health selection effect. Moreover, while the level of medical care in cities is relatively high, which can prolong life, most people live with diseases without being able to significantly improving their level of living. Additionally, older adults who regularly exercise outdoors are more capable of self-care [11, 26, 28].

There is significant evidence that depression among the elderly in many countries is on the rise, and it is significantly associated with impairments in ADLs and IADLs [31, 32]. The negative impact of declining ADLs on older adults' mental health can potentially be mitigated by better social networks [33]; the presence of neighborhood resources in the community for older adults in China is an important protective factor for the mental health of older adults, and may reduce the effects of depressive conditions on declining ADLs. A fall in the elderly may result in a decline in physical, psychological, or social function, while a severe fall may result in impaired daily living abilities and even disability as a result of the fall [34, 35]. ADLs and IADLs were negatively affected by falls in older adults in this study, which examined whether falls occurred in the past year. Accordingly, it would be worthwhile to explore and analyze the risk factors and the protective factors for falls in older adults.

Consistent with previous studies, the number of chronic diseases significantly affected ADL and IADL. In this study, taking no chronic disease as a reference, the number of chronic diseases increased, and the OR increased accordingly, with a trend test result of *p* < 0.001. Age had a significant impact on ADL. In addition, the influence of age on the ability to perform daily activities was observed. Taking the lower age group as a reference, the OR value increased as the age group increases, and the trend test *p* < 0.001. Therefore, it is imperative that primary healthcare providers pay special attention to older adults and improve screening for multimorbidity and follow-up needs.

Although this study was a nationwide large-scale survey, it still had the following limitations. First, causal analysis could not be performed based on cross-sectional data due to inherent defects. Second, because all chronic diseases were self-reported, it was difficult to determine the awareness rate of the respondents. However, the survey indicates that 67.4% of the respondents underwent physical examinations annually; therefore, these results were deemed somewhat reliable. Third, owing to the large amount of missing data for some chronic diseases, scale data and variables, it was not possible to include all chronic diseases, ADL, IADL, and CESD-10 scores in this study. Furthermore, the low percentage of senior citizens in the population included in the analysis, and the even lower knowledge of chronic diseases in this group, contributed to the instability of the regression model.

## Conclusion

It is believed that the incidence of chronic diseases and multimorbidities increases with age. Nevertheless, the CLHLS study indicated that after peaking between 75 and 84 years of age, the incidence gradually declined. It is believed that the age group of 75–84 years is critical for the longevity of the older adults. With an increasingly aging population, it is imperative to make more efforts to coexist with chronic diseases. Having regular medical check-ups can be done by the government to detect potential risks of incapacity in a timely manner and prevent them from occurring, and thus make it easier for the elderly to take care of themselves on a daily basis. Moreover, we recommend moderate exercise, increasing cultural knowledge, enjoying nature, avoiding being obese, and reducing chronic diseases, which will help to steadily improve the health of older adults and promote healthy aging.

## Data Availability

The CLHLS questionnaires are available at https://sites.duke.edu/centerforaging/programs/chinese-longitudinal-healthy-longevity-survey-clhls/survey-documentation/questionnaires/. The full datasets used in this analysis are available from the corresponding author upon reasonable request.

## Abbreviations

BMI: Body Mass Index
ADL: Activities of Daily Living
IADL: Instrumental Activities of Daily Living
CVD: Cardiovascular Disease
